# Digits-in-Noise Test as an Assessment Tool for Hearing Loss and Hearing Aids

**DOI:** 10.3390/audiolres14020030

**Published:** 2024-04-08

**Authors:** Carly Schimmel, Kayla Cormier, Vinaya Manchaiah, De Wet Swanepoel, Anu Sharma

**Affiliations:** 1 Department of Speech, Language, and Hearing Sciences, University of Colorado Boulder, Boulder, CO 80309, USA; carly.schimmel@colorado.edu (C.S.); kayla.cormier@colorado.edu (K.C.); 2 Department of Otolaryngology-Head and Neck Surgery, University of Colorado School of Medicine, Aurora, CO 80045, USA; vinaya.manchaiah@cuanschutz.edu (V.M.); dewet.swanepoel@up.ac.za (D.W.S.); 3 UCHealth Hearing and Balance, University of Colorado Hospital, Aurora, CO 80045, USA; 4 Virtual Hearing Laboratory, Collaborative Initiative between University of Colorado School of Medicine and University of Pretoria, Aurora, CO 80045, USA; 5 Department of Speech-Language Pathology and Audiology, University of Pretoria, Pretoria 0002, South Africa; 6 Department of Speech and Hearing, School of Allied Health Sciences, Manipal Academy of Higher Education, Manipal 576104, India

**Keywords:** speech-in-noise, over-the-counter hearing aids, teleaudiology

## Abstract

The aim of this study was to examine the relationship between an American English Digits in Noise (DIN) test and commonly used audiological measures to evaluate the DIN test’s ability to detect hearing loss and validate hearing aid fitting. QuickSIN and DIN tests were completed by participants with untreated hearing loss (*n* = 46), prescription hearing aids (*n* = 15), and over-the-counter (OTC) hearing aids (*n* = 12). Performance on the QuickSIN showed moderate positive correlations with DIN for untreated hearing loss participants and prescription hearing aid users, but not for OTC hearing aid users. For untreated hearing loss participants, both QuickSIN and DIN tests showed positive moderate to strong correlations with high frequency puretone averages. In OTC users, DIN scores did not significantly change over a 6-month time period and were better when conducted remotely compared to in-person testing. Our results suggest that the DIN test may be a feasible monitoring option for individuals with hearing loss and those fitted with hearing aids. However, due to small sample size in this pilot study, future research is needed to examine DIN test’s utility for fitting and validating OTC hearing aids.

## 1. Introduction

Recent studies have emphasized the importance of early identification and treatment of hearing loss in adults due to the compensatory brain changes that occur even in its early stages [1,2,3,4,5,6]. Despite the potential benefits of hearing aids in reversing these changes and improving cognition [1,2,3], a significant global gap exists in hearing aid usage [7,8]; approximately 83% of the 401.4 million individuals who could benefit from them do not use hearing aids [7]. This gap is attributed to various factors, including perceived need, financial constraints, and stigma [9,10,11]. To overcome some of these burdens and challenges, there is a move towards remote care and decentralized hearing healthcare to improve efficiency and access to hearing healthcare needs [11,12]. Such an approach requires validated tools that are widely accessible and available over the internet for self-administration.

Furthermore, with the recent passing of the Over-the-Counter (OTC) Hearing Aid Act of 2017 [13] and consequently the gained popularity of OTC hearing aids, more people may be curious about pursuing hearing aids with increased accessibility and lower price compared to prescription hearing aids fit by a hearing healthcare professional (HHP). However, these devices do not require a formal hearing test. Therefore, it is important to establish fitting and validation methods to remotely assess hearing and speech perception difficulties, in particular for those pursuing OTC hearing aids.

Additionally, remote validation of prescription hearing aids may also be important. Several factors, such as geographic isolation, economic barriers, and limited access to healthcare professionals can impede frequent in-person consultations for patients or consumers [14]. Lower income individuals face additional barriers including lack of transportation, inability to take time off work, inadequate insurance coverage, and the financial burden of appointment copays [14,15]. In these cases, digital technology becomes an important tool in the modern healthcare landscape. By utilizing mobile and internet-based assessment, monitoring, and treatment options, hearing healthcare can become more accessible and inclusive.

Mobile and internet-based technologies are now able to offer ways to remotely deliver useful assessments such as speech-in-noise evaluations. Understanding speech in the presence of noise is a common complaint among people who have hearing loss and difficulties with speech perception in noise may be more indicative of real-world functional difficulties than the standard hearing test measure of detection of puretones alone [16,17,18]. Speech perception in noise testing is often done clinically at a hearing test or hearing aid consultation appointment to identify a patient’s difficulties in real-world environments due to their hearing loss. Some audiologists may also perform aided speech in noise testing at follow up appointments to examine hearing aid benefit, although it is less common. A commonly used clinical speech perception in noise test is the QuickSIN [19], which is a sentence-based measure in which patients repeat back sentences in varying levels of background noise. Testing speech-in-noise is a helpful tool in determining the relevant impact of hearing loss in a patient’s life and is a test that can be used to compare performance with and without hearing aids to examine hearing aid benefit.

Alternatively, the Digits in Noise (DIN) test is a self-administered speech-in-noise test that can be delivered over telephone, internet or app-based using a variety of headphones. It presents digit triplets (0–9) in the presence of broadband noise and is scored as a speech reception threshold (SRT). The test is efficient and typically takes less than 5 min to complete [20]. The DIN test was originally developed in Dutch and created to be used via telephone [21,22,23]. Currently, it has been validated as a test to take on a smartphone or web-based via the internet, including in personal home settings, and is available in several languages including American English [23,24,25]. The DIN test allows for accurate and reproducible thresholds that can be presented though a digital platform with minimal linguistic load [20,24,25,26].

The DIN test has shown robust correlations with audiometric puretone averages in several studies [20,21,23,26,27,28,29,30]. Additionally, previous studies have shown significant correlations with the DIN test and other sentence- and word-based speech perception in noise tests in those with normal hearing and hearing loss [20,27,31,32,33]. A study by Jansen and colleagues (2012) compared the Dutch DIN test with a sentence in noise task, finding strong correlations between the speech-in-noise tests for those with normal hearing, prescription hearing aids, and cochlear implants [27]. In addition to these strong correlations, the closed set of digits reduces the linguistic complexity and memory demands that are more typical of sentence-based tests. Therefore, the DIN test may be more broadly appropriate for a wider range of populations, such as children and second language learners [20].

To our knowledge, no studies have compared the American English DIN test with the QuickSIN clinical test, which is commonly used by HHPs in evaluating hearing loss in the clinic. As the QuickSIN test is the more commonly used clinical speech-in-noise test, it is important to know how the DIN and QuickSIN tests compare to each other as a measure for evaluating both the presence of hearing loss and hearing aid performance. Not only does the DIN test have implications as a hearing loss screening tool, which may be of particular interest for those pursuing OTC hearing aids, but it may also be useful in the fine tuning and validation of prescription hearing aid fittings. The overall aim of this study was to examine the relationship between DIN and other commonly used audiological measures in individuals with hearing loss, prescription hearing aid users, and OTC hearing aid users to evaluate its use to diagnose and treat hearing losses. To assess this, we aimed to examine: (1) the relationship between DIN, QuickSIN, word recognition in quiet, and puretone averages in untreated and treated hearing loss; (2) the use of DIN as a remote hearing assessment tool in OTC hearing aid users; and (3) changes in DIN longitudinally in OTC hearing aid users.

## 2. Materials and Methods

This pilot study examined speech-in-noise abilities in those with untreated hearing loss and hearing aid users. Three different groups of participants were tested, including (a) those with hearing loss who did not wear hearing aids, (b) those who used prescription hearing aids, and (c) those who used OTC hearing aids. The data was collected as part of a series of larger studies examining the impacts of hearing aids on neuroplasticity, cognition, and audiologic behavioral outcomes. All in-person laboratory data was collected in a sound attenuated booth in the Brain and Behavior Laboratory at the University of Colorado Boulder. Participants were recruited through a variety of methods including social media flyers, paper flyers, community health fairs and outreach, and word of mouth. The protocol (18-0087) was approved by the Institutional Review Board at the University of Colorado Boulder. All subjects provided written informed consent prior to participation in the study.

### 2.1. Participants

All participants spoke fluent English, reported normal or corrected to normal vision, and denied the presence of neurological disorders. Audiometry was completed using a GSI-61 (Grayson-Stadler, Eden Prairie, MN, USA) audiometer with ER-3A (Etymotic, Elk Grove Village, IL, USA) insert earphones or TDH-39 (Telephonics, Farmingdale, NY, USA) supra-aural headphones. Commonly used ways to report air conduction hearing thresholds were used to summarize audiometric findings for analysis, which included a three-frequency average of hearing thresholds at 500, 1000, and 2000 Hz (i.e., PTA-3), a four-frequency average at 500, 1000, 2000, and 4000 Hz (i.e., PTA-4), and a high frequency average at 3000, 4000, and 6000 Hz (i.e., PTA-HF). Given that all participants had bilateral hearing loss, audiometric thresholds were averaged between the two ears and used for subsequent analyses, as has been done in prior research comparing puretone thresholds and speech perception in noise [34].

**Figure 1:**
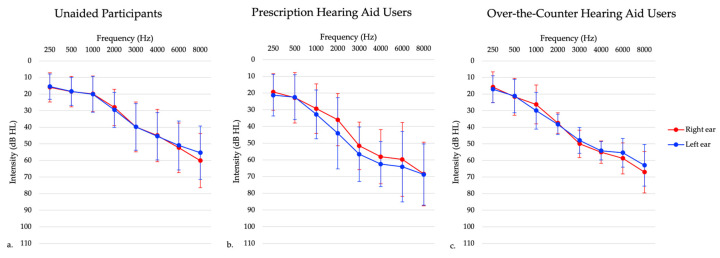
Average audiogram of participants with (**a**) untreated hearing loss, (**b**) prescription hearing aids, and (**c**) over-the-counter hearing aids. This graph shows right ear (red) and left ear (blue) thresholds separately.

Participants fell into three different groups, described further below: individuals with untreated hearing loss, traditional hearing aid users, and OTC hearing aid users. Adults with unilateral hearing loss and conductive hearing loss were excluded from the study in all three groups. Prescription and OTC hearing aid users had hearing aid fittings measured using NAL-NL2 prescriptive targets [35] utilizing an Audioscan Verifit probe-microphone verification system (Audioscan, Dorchester, ON, Canada). The root mean square error (RMSE) was calculated for hearing aid fittings to measure the accuracy of hearing aid amplification to the NAL-NL2 targets. RMSE was averaged across 500, 1000, 2000, and 4000 Hz [36]. Then the RMSE values from each ear were averaged to create one binaural value. Hearing aids are considered to be properly fit if the RMSE is within 5 dB RMS [36]. Average RMSE values for each hearing aid group can be seen in Table 1. Average RMSE and age were not significantly different in the prescription hearing aid users and OTC hearing aid users, as determined using an independent samples *t*-test (*p* > 0.05). Table 1 provides participant characteristics and Figure 1 presents average hearing thresholds.

#### 2.1.1. Experiment 1: Individuals with Untreated Hearing Loss

Forty six participants in this group presented with bilateral sensorineural hearing loss (see Figure 1), and had no prior experience using hearing aids. Inclusion criteria included adults aged 20–85 years old and those with bilateral sensorineural hearing loss. Exclusion criteria included a diagnosed neurological condition, non-English speaking (since they needed to speak English to participate in the clinical tests), and unilateral and conductive/mixed hearing losses. Those with asymmetrical hearing losses were included in this study to best represent the general hearing loss population, defined as a high frequency puretone average (3, 4, 6 kHz) difference between ears of 15 dB or greater [37]. In this sample, one participant presented with a high frequency puretone average asymmetry, with a maximum asymmetry of 32 dB HL. The maximum asymmetry at any given threshold in the 3–6 kHz range was 35 dB HL. Participants completed testing at one session only.

#### 2.1.2. Experiment 2: Prescriptions Hearing Aid Users

Sixteen participants in this group were experienced hearing aid users (i.e., using hearing aids for 6 months or longer) who presented with bilateral sensorineural hearing loss (see Figure 1). One participant was excluded due to their age at the time of testing being greater than three standard deviations from the mean age. Inclusion criteria included adults aged 20–85 years old and those who have been wearing hearing aids bilaterally for six or more months. Exclusion criteria included a diagnosed neurological condition, non-English speaking, and those with unilateral and conductive/mixed hearing losses. Those with asymmetrical hearing losses were included in this study to best represent the hearing aid user population. In this sample, two participants presented with a high frequency puretone average asymmetry, with a maximum asymmetry of 55 dB HL. Participants completed testing at one session only.

#### 2.1.3. Experiment 3: Over-the-Counter (OTC) Hearing Aid Users

A subset of 13 participants from the untreated hearing loss participants received FDA-approved self-fitting OTC hearing aids that were self-fit via a smartphone app. These hearing aids were from different companies but were only either behind-the-ear or receiver-in-the-canal style hearing aids. These participants were followed longitudinally for approximately 6 months. All participants presented with symmetrical bilateral mild to moderate sensorineural hearing loss, as demonstrated in Figure 1. These participants had no prior experience with hearing aid use. Inclusion criteria for this study included adults aged 20–85 years old and mild to moderate high-frequency sensorineural hearing loss, defined as hearing thresholds of 25–70 dB HL between 2000–4000 Hz. Inclusion criteria was chosen to reflect intentions of OTC hearing aid policies of indication for individuals with perceived mild to moderate hearing loss. Exclusion criteria included those with unilateral hearing losses, conductive/mixed hearing losses, prior hearing aid use, a diagnosed neurological condition, and non-English speaking. In this sample, there were no participants with asymmetric hearing losses. One participant withdrew from the study following one month of hearing aid use. Participants completed testing in the laboratory setting at baseline, which was defined as within 5 days of receiving their hearing aids and returned approximately 1 ± 0.07 month (mean ± SEM), 4 ± 0.07 months, and 6 ± 0.07 months following hearing aid use. Two participants did not complete all the laboratory setting testing. One participant missed the four-month follow-up visit, while another participant missed the six-month follow-up visit.

### 2.2. Speech Intelligibility Testing

For participants with untreated hearing loss, speech intelligibility testing in quiet was evaluated using word recognition score (WRS) testing in quiet. In WRS, the scores can range from 0 to 100% with higher scores indicating better performance. Ear-specific WRS scores were not significantly different and were averaged for analysis.

In all participants, auditory-only speech-in-noise perception was evaluated using the QuickSIN test, a clinically validated measure of auditory speech perception in background noise [19]. Participants completed this testing seated in a sound booth in front of a speaker at 0° azimuth with speech presented at 65 dB SPL. Testing was completed binaurally through speakers to allow for testing in the aided hearing aid condition for those groups who wear hearing aids, as this is how testing is completed clinically in order to compare to normative values for the QuickSIN and DIN tests [19]. Participants repeated sentences in background noise. They were presented with two randomized lists of 6 sentences each. Each sentence was presented in the context of multi-talker background noise ranging from 0 to 25 dB signal-to-noise ratio (SNR). The test is scored in terms of key words correct and a computation is performed to determine the dB SNR loss, which is referenced to normal hearing listeners. On the QuickSIN test, a lower score indicates better speech perception in noise abilities. Clinical categories for the QuickSIN test provided by Etymotic Research are as follows: 0–3 dB SNR indicates normal/near normal, 3–7 dB SNR indicates a mild SNR loss, 7–15 dB SNR indicates a moderate SNR loss, and greater than 15 dB SNR indicates a severe SNR loss [19].

In all participants, auditory-only speech-in-noise perception was also evaluated using the American English Digits in Noise (DIN) test via the hearDigits web-based application developed by the hearX Group (Pretoria, South Africa) [26]. Participants were seated in a sound booth and completed an automated, self-administered, web-based DIN test on an iPad tablet. Like the QuickSIN test, the DIN test was completed in the soundfield through the iPad speakers. As stated above, this presentation method allowed for participants to complete testing in the aided hearing aid condition. Participants first heard three numbers repeatedly and were asked to set the volume of those numbers to a comfortable listening level. Participants were told to think of this as the volume they would set their TV or radio at. Following this, participants were required to select digit triplets (all digits 0–9 included) heard in the presence of a broadband speech-shaped background noise. The level of the background noise changed adaptively throughout testing. A total of 23 trials were completed. Following the trials, a computation was performed to determine the dB SNR required for the participant to get 50% of trials correct based on the adaptive changing of the background noise. On the DIN test, a lower score indicates a better performance of speech perception in noise. Previous research has determined normal performance in noise, which has been analyzed in listeners with normal hearing and hearing loss using the American English DIN [38]. For participants who wore prescription or OTC hearing aids, all speech-in-noise testing was completed aided, with hearing aids on.

Individuals with untreated hearing loss completed the DIN in the unaided condition. Prescription and OTC hearing aid users completed the DIN in the aided condition only. In addition, participants who received OTC hearing aids were also asked to take the DIN test remotely at home while utilizing their hearing aids on approximately a bimonthly basis. Participants were emailed reminders with the link to complete at-home DIN testing on any device in which they could access the internet. This was the same link as used for in person testing. Written instructions asked participants to complete this test utilizing their device speakers (i.e., phone, computer, or tablet) with their hearing aids set to their typical everyday settings in a quiet environment. The remaining written instructions were the same as detailed above.

### 2.3. Statistical Analysis

Prior to statistical analyses, the data were first visualized to identify outliers. No outliers were identified. The Shapiro-Wilk Test and quantile-quantile plots confirmed assumptions of normality. To examine the relationship between DIN and commonly used audiological measures, Pearson correlations were examined between speech-in-noise measures (QuickSIN and DIN tests) separately for each of the three groups. For individuals with untreated hearing loss both speech-in-noise measurements (QuickSIN and DIN tests) were also correlated with puretone averages. Additionally, speech-in-noise measurements were correlated with measures of speech reception abilities in quiet (WRS) in this population. Strength of correlation coefficients are defined as weak (*r* = 0.1–0.39), moderate (*r* = 0.4–0.69), or strong (*r* = 0.7–0.89) [39].

Additionally, to examine DIN remotely and over time in OTC hearing aid users, a multilevel analysis (MLM) with a random intercept was utilized to examine changes in DIN over time. Time was coded as months of hearing aid use and was rounded to the nearest month. Contrast codes were utilized to distinguish between DIN scores collected in the laboratory environment and those obtained remotely in the at-home environment. The interaction between time and test environment was also included in the model.

## 3. Results

### 3.1. Experiment 1: Individuals with Untreated Hearing Loss

**Figure 2:**
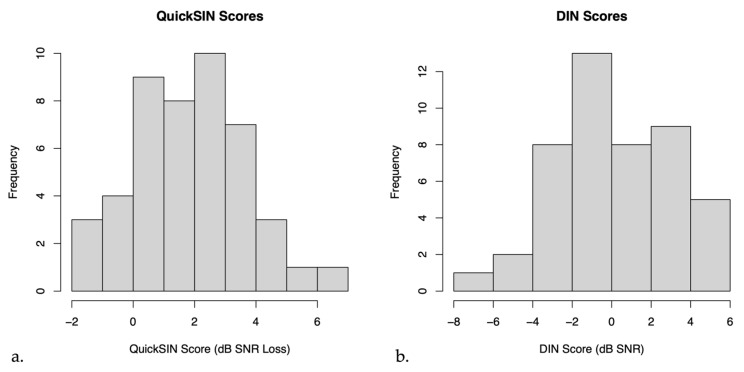
Histograms of (**a**) QuickSIN scores and (**b**) Digits in Noise (DIN) scores in untreated hearing loss participants.

On average, the mean QuickSIN score for untreated hearing loss participants was 2.13 ± 0.28 dB SNR loss (mean ± SEM), indicating near normal hearing in noise. The mean DIN score for these participants was 0.20 ± 0.45 dB SNR (mean ± SEM), indicating on average reduced SNR scores compared to normal hearing persons [38]. Figure 2 below shows a histogram of the distribution of QuickSIN and DIN scores.

**Figure 3:**
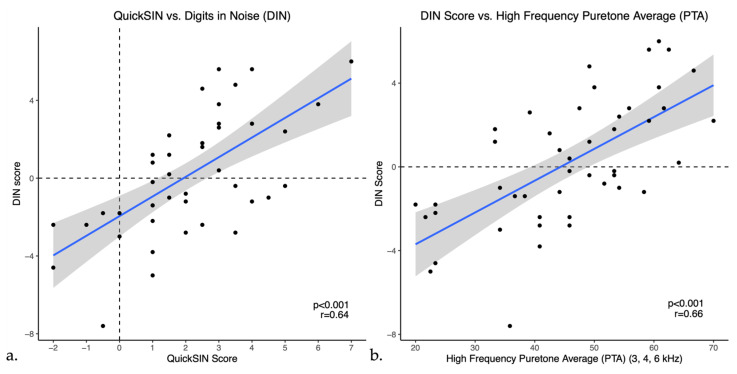
Scatter plots of Digits in Noise (DIN) scores versus (**a**) QuickSIN and (**b**) High frequency PTA (PTA-HF) in untreated hearing loss. The blue line represents the linear regression line and black dots indicate individual data points.

Pearson correlations indicated that DIN scores showed a moderate positive correlation with QuickSIN scores (*r* = 0.64, *p* < 0.001) as shown in Figure 3a. DIN scores also showed weak to moderate positive correlations with bilateral puretone averages, correlating most strongly with the PTA-HF (*r* = 0.65, *p* < 0.001), shown in Figure 3b. Furthermore, DIN scores had a moderate negative correlation with word recognition scores in quiet (*r* = −0.53, *p* < 0.001), indicating that a higher percentage on word recognition in quiet was correlated with a lower or better SNR in noise. Moreover, similar to the DIN test, QuickSIN scores had weak to moderate positive correlations with all puretone averages as well as a moderate negative correlation with word recognition in quiet (WRS) as illustrated in Table 2. To ensure accuracy and stability of the results, we examined the data removing the one participant with asymmetric hearing loss and this does not change the results.

### 3.2. Experiment 2: Prescription Hearing Aid Users

**Figure 4:**
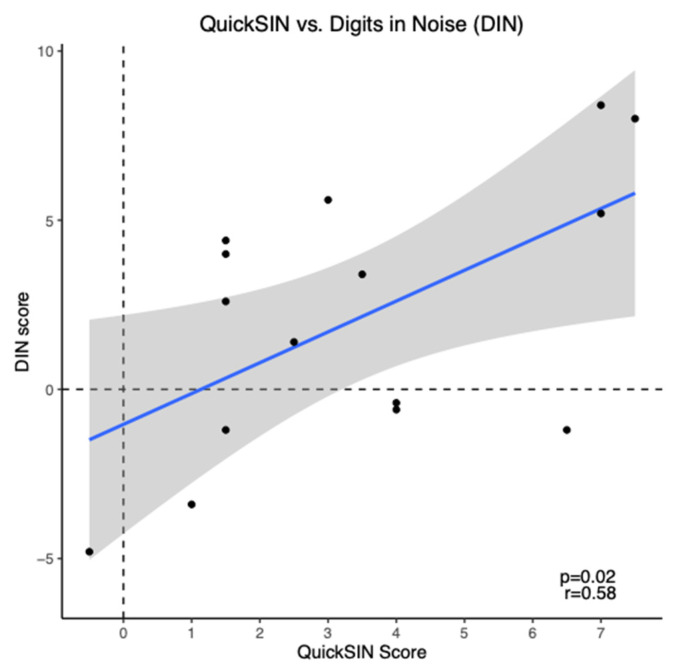
Scatter plot of Digits in Noise (DIN) scores versus QuickSIN scores for prescription hearing aid users (*n* = 15). The blue line represents the linear regression line and black dots indicate individual data points.

On average, the mean aided QuickSIN score for prescription hearing aid users was 3.43 ± 0.65 dB SNR loss (mean ± SEM), indicating a mild SNR loss. The mean aided DIN score for these participants was 2.1 ± 1.03 dB SNR (mean ± SEM), indicating reduced SNR scores compared to normal hearing persons [38]. DIN scores showed a moderate positive correlation with QuickSIN scores (*r* = 0.58, *p* = 0.02) as shown in Figure 4. To ensure accuracy and stability of the results, we examined the data removing the two participants with asymmetric hearing loss and this does not change the results.

### 3.3. Experiment 3: Over-the-Counter Hearing Aid Users

On average, at all in-person visits, OTC hearing aid users demonstrated QuickSIN scores within the normal/near normal hearing range (see Table 3). Average in-person DIN scores were all above normal hearing SRTs [38], as demonstrated in Table 3.

At baseline, QuickSIN scores did not have a statistically significant correlation with DIN scores in this population (*r* = −0.17, *p* = 0.587). In addition, following six months of hearing aid use, QuickSIN and DIN scores continued to show no significant correlation (*r* = −0.01, *p* = 0.980). Neither DIN scores nor QuickSIN scores had a significant change over time.

#### DIN Testing Remotely Versus In-Person

Participants were additionally asked to complete DIN testing at home while utilizing their OTC hearing aids. A total of 10 participants completed this testing at least once over the course of their 6 months in the study. Two participants completed no at-home DIN testing, three participants completed at-home DIN testing once, five participants completed at-home DIN testing twice, and two participants completed at-home DIN testing three times. On average, at each visit DIN scores indicated reduced SNR scores compared to normal hearing persons [38].

**Figure 5:**
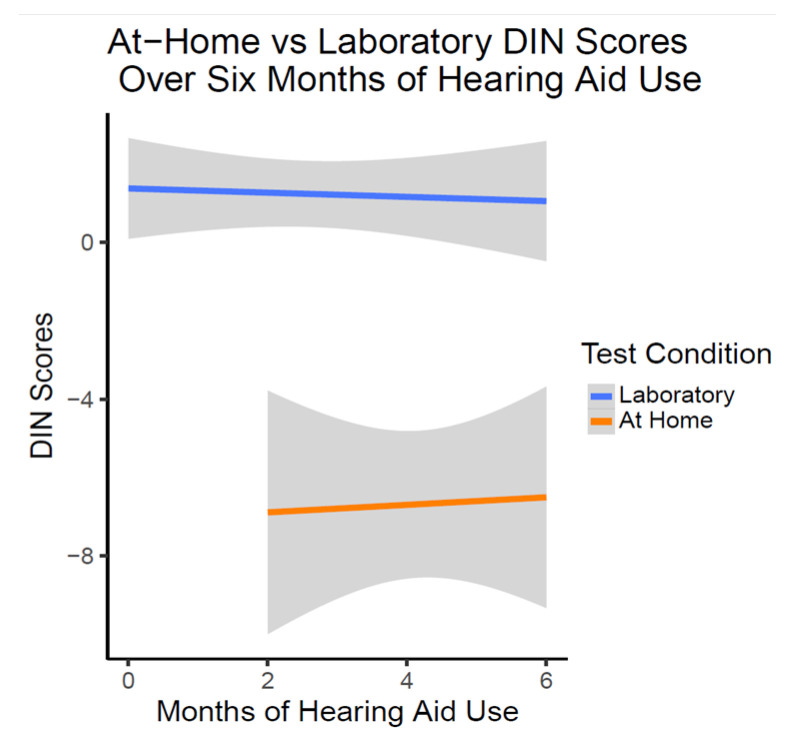
DIN Scores over six months of OTC hearing aid use (*n* = 10). This figure shows the lack of change in DIN scores with OTC hearing aid use. On average, at-home DIN scores were better than DIN scores obtained in the laboratory condition at all time points.

There was no significant change in laboratory in person or at-home DIN scores over six months of hearing aid use (*t*(54.67) = 0.961, *p* = 0.341). As illustrated in Figure 5, at-home DIN scores were estimated on average to be significantly lower (better) than DIN scores obtained in the laboratory setting as shown with linear trajectory lines (*t*(54.36) = −5.63, *p* < 0.001). This relationship of the at-home DIN scores being better did not change over the six months of hearing aid use (*t*(53.76) = 0.903, *p* = 0.370).

## 4. Discussion

The current study evaluates the applicability of American English DIN in the assessment of hearing loss and hearing aids. The relationship between DIN and commonly used audiological measures are examined. The following section discusses the key findings.

### 4.1. Experiment 1: Individuals with Untreated Hearing Loss

This study found the DIN significantly correlates with the clinical QuickSIN test in those with untreated high frequency sensorineural hearing loss. This indicates that the DIN test may be a good measure of speech perception in noise in situations without clinician involvement. This correlation was found despite differences in speech material (digits versus sentences), in background noise maskers (speech-shaped broadband noise versus multitalker babble), and presentation levels (65 dB SPL versus a comfortable listening level). This finding is consistent with previous studies correlating DIN tests with sentence- and word-based speech perception in noise testing [20,23,27,31,32,33].

Additionally, the DIN test shows a moderate positive correlation with puretone averages (PTA) consistent with previous studies [20,21,23,26,27,28,29,30]. This correlation was strongest with a PTA-HF. This finding is of particular importance when considering that age-related hearing loss typically impacts higher frequencies first. These results indicate that the DIN test may be useful as a screener for hearing loss to estimate hearing levels and speech perception in noise difficulties [20,21,23,26,27,28,29,30,31,32,33]. Given that OTC hearing aids do not require a clinical hearing test prior to purchase, the results of this study indicate that the DIN test may be a viable remote option for these individuals to aid screening for hearing loss and in the determination to acquire hearing aids. Furthermore, these results indicated that as the DIN test becomes more widely available, hearing healthcare professionals can feel reassured of the clinical comparability of this remote test. At-home DIN testing may result in individuals seeking a formal hearing test, so hearing healthcare professionals should be familiar with the test. Additionally, in clinical settings in which the linguistic needs of the QuickSIN test are too demanding, our results suggest that the DIN test could be a viable alternative to complete speech-in-noise testing in these patients [20].

In this experiment, we also do not exclude those with asymmetric hearing losses in order to best represent the general hearing loss population. This population may also be an important untreated hearing loss population for OTC hearing aids to capture. Many individuals with asymmetric hearing losses choose to only use one hearing aid despite having bilateral hearing loss, with some of these cases citing economic reasons for this choice [40]. However, research has shown better outcomes for those with asymmetric hearing losses when bilaterally aided, including better speech perception in noise performance and satisfaction with hearing aids [40,41]. Those with asymmetrical hearing losses may choose to pursue OTC hearing aids, which are often sold in pairs, due to more ease of purchase and cheaper cost, and this may help to provide those individuals with earlier adaptation to bilateral fitting.

### 4.2. Experiment 2: Prescription Hearing Aid Users

This study shows, in participants who were long-term prescription hearing aid users (i.e., utilizing hearing aids for at least 6 months), QuickSIN and DIN scores were significantly correlated, consistent with previous studies in hearing aid users using testing in languages and dialects other than American English [26,29,32]. Kaandorp and colleagues measured the Dutch DIN test and a standard Dutch sentence in noise test in 12 participants with normal hearing, 24 participants with hearing aids, and 24 participants with cochlear implants [32]. They found that the DIN test was more feasible compared to the sentence test and that the two tests were highly correlated in the group of normal hearing participants and hearing aid users (*r* = 0.95). While the correlations reported in this present study were not as high as Kaandorp and colleagues, our participants were on average older (mean age of 71 years compared to 59 years) which may have contributed to differences in speech understanding [32]. Furthermore, when comparing the PTA of the study samples, when looking at a PTA using 1,2, and 4 kHz, our study sample had an average PTA of 44 dB HL compared to the study by Kaandorp et al. which was 59 dB HL. The Dutch sentence in noise task used finds an SNR-50 using an adaptive method and sentences are presented in speech-shaped noise. This may be another reason for their higher correlation as the DIN is also adaptive and presented in speech-shaped noise, whereas the QuickSIN uses fixed SNR and a multitalker babble background noise.

Other studies have looked at the DIN in prescription hearing aid users, but for a different purpose such as comparing unaided and aided scores. Potgieter, Swanepoel, and Smits investigated the South African DIN test in nine adult hearing aid users (mean age of 72 years) [26]. These participants took the DIN test both aided and unaided, presented from speakers in a sound booth via a smartphone app. They also completed word recognition testing. On average, participants had a dB SNR score of −7.2 while aided, which provided a 0.8 dB SNR improvement compared to their unaided condition. While the sample from Potgieter, Swanepoel, and Smits [26] was closer in age and sample size to our study participants, their participants did on average show better scores on the DIN test (−7.2 dB SNR) compared to our study sample (2.1 dB SNR). In their study, the DIN was presented through free-field speakers connected to the smartphone app, so it is possible that their method of testing created clearer sound quality as compared to our study in which the DIN was presented through the iPad tablet speaker. Additionally, in the present study, the DIN was part of a long test battery and users may have had less concentration compared to other studies in which DIN was the focus.

It is possible that the correlation between DIN and QuickSIN in our study is lower than other studies comparing DIN with speech testing due differences in frequency bandwidth of the tests. Monson and Buss analyzed several different speech stimuli, including QuickSIN, NU-6 word recognition, HINT and several more [42]. For the QuickSIN, they found that stimuli had a steep roll off after 8 kHz, indicating that stimuli did not include higher frequencies that are important for speech understanding in noise. Alternatively, DIN testing may be able to access a higher frequency bandwidth past 8 kHz [43].

Our data indicates that while there is some variation in the DIN test depending on the methods used, overall the DIN test may be a good measure of validation for prescription hearing aid fittings, which patients could utilize independently or in the clinic. However, the correlation for prescription hearing aid users was not as strong compared to the untreated hearing loss results. It is likely that the small sample size of individuals in this group contributed to the weaker correlation results. Therefore, future research may benefit from examining hearing aid users and non-hearing aid users separately and examining results with and without hearing aids (i.e., measure of benefit). 

### 4.3. Experiment 3: Over-the-Counter (OTC) Hearing Aid Users

#### 4.3.1. Relationship of DIN and QuickSIN

In participants utilizing OTC hearing aids, we did not see any correlations between the DIN and QuickSIN tests at baseline or after 6 months of hearing aid use. A previous study by De Sousa and colleagues investigated the QuickSIN and DIN tests in self-fitted hearing aid users, however this study did not compare aided correlations but instead examined benefit scores for these tests in comparison to audiologist fit hearing aid users [44]. In a randomized clinical effectiveness trial examining the effectiveness of OTC devices with audiologist fit devices, 68 adults with self-perceived mild to moderate hearing loss trialed hearing aids over a 6-week period. The OTC group trialed devices without support or access to fine tuning for 2 weeks and were then assessed again after 4 additional weeks with access to support and fine tuning. The audiologist fit group had access to support and hearing aid adjustments remotely with an audiologist as requested. At the end of their 6-week trial, this study found no differences between the groups in self-reported hearing aid benefit (as measured by the APHAB and International Outcome Inventory for Hearing Aids (IOI-HA)) or speech perception in noise scores measured by the DIN and QuickSIN tests. It is important to consider that participants in their study had varied levels of support and had a shorter duration of hearing aid use (6 weeks), whereas our study was conducted over 6 months. Additionally, this study had a larger sample size (*n* = 68) compared with the current study which only includes 12 participants.

#### 4.3.2. Remote versus In-Person Testing for OTC Hearing Aids

In the present study, when participants independently tested with the DIN remotely at home, this was not correlated with their in-person DIN scores at any time point over the first six months of hearing aid use. In fact, their remote scores on average were significantly better than when tested in ideal conditions in the sound booth in our laboratory. The at-home testing scores were in a range more typically expected for the DIN test (i.e., [38,43]), indicating important differences in testing environments. While it is unknown what type of home environments the participants were in when independently completing the test, it is possible that in their home environment, participants were more comfortable with testing and did not feel as much pressure as compared to the laboratory setting where experimenters were present. It is also possible that there may be an effect of presentation device in that there may be differences whether the DIN is administered through a phone, tablet or computer. In the laboratory setting, all participants in this study completed the test on the same iPad tablet, however it is unknown what devices were used by the OTC hearing aid users when taken independently at home. Additionally in the laboratory setting, participants were completing several tests in addition to the DIN test. It is possible that fatigue could have played a role in the higher (worse) DIN scores obtained in the laboratory setting. Further research into fatigue and time of day testing is warranted for the most appropriate guidance to be provided to individuals using the DIN test.

In studies of remote versus in-person testing in other domains, Hoge and colleagues [45] found that while episodic and working memory scores were not significantly different between in-person or remote testing, they had poor to moderate agreement unless removing participants who may have invalid scores due to reasons such as internet connectivity disruption. They also found that participants demonstrated better chair stand physical performance in remote testing, possibly due to access to more cushioned chairs at different heights compared to the chairs used in in person testing. Similar to the study by Hoge and colleagues [45], it is possible that at home testing equipment may have made a difference in results. Our participants had the option to take the DIN on any device that could access internet, so it is possible that there were sound quality differences compared to the iPad tablet used in our in-person laboratory setting that resulted in better scores at home. Given that the DIN test is primarily meant for remote testing at home, it would be useful for larger sample studies to corroborate this finding.

#### 4.3.3. Speech Perception in Noise Testing Longitudinally in OTC HA Users

The present study did not find any changes or improvements in the QuickSIN or DIN scores over time in OTC hearing aid users. The current literature on this is mixed. Some previous studies have seen significant improvements in speech perception in noise scores (using the QuickSIN test) after six months of hearing aid use [1,46]. Other studies have not seen clear differences in speech perception in noise over time with hearing aid use, implying that there may be suprathreshold hearing and central auditory processing deficits that are not addressed by treatment with hearing aids [47,48,49]. In a study by Dawes and Munro [47], new hearing aid users were followed for 30 days after their hearing aid fitting and compared to a control group of 20 experienced hearing aid users, in which no improvement in speech perception in noise scores was found between groups as a whole [47]. However, there was a greater improvement in a subset of new hearing aid users who had hearing loss greater than 40 dB HL and wore their hearing aids at least six hours per day. Glick and Sharma (2020) also noted an improvement in QuickSIN scores after six months of hearing aid use for participants wearing hearing aids a minimum of five hours per day [1]. Therefore, hours of hearing aid use may be an important factor when examining speech-in-noise changes over time in hearing aid users. In the present study we were unable to objectively monitor OTC hearing aid use and it is possible that subjects were wearing their hearing aids less frequently than reported. Self-reported hours of hearing aid use are not always reliable, with individuals frequently overestimating their hours of use [50,51]. Additionally, similar to the study by Dawes and Munro [47], our sample of OTC hearing aid users demonstrated a wide range in daily hearing aid use (0.17–13.55 hours/day) and it is possible that there may be differences in speech perception over time with more consistent hearing aid use. Average reported daily hours of hearing aid use was similar to a previous study by Swanepoel and colleagues in 2023 which investigated differences in self-reported hours of usage when comparing OTC hearing aid users and those fit traditionally by a hearing healthcare professional and found that traditional hearing aid users reported significantly longer hours of daily use compared with the OTC hearing aid users [52]. It is important to note that OTC hearing aid users may have different expectations regarding the amount of daily hearing aid use compared to prescription hearing aid users, such that they may view their devices as needing to be used more situationally rather than regularly, akin to reading (OTC) glasses. Therefore, more research is needed to establish how hearing aid benefits may vary as a function of amount of daily hearing aid use.

It’s possible that the DIN test could be a potential validation tool for those with OTC hearing aids, however in our study it only correlated to clinical measures (i.e., the QuickSIN test) for prescription hearing aid users. For the OTC HA group, neither in-person DIN testing nor remote testing correlated with QuickSIN scores. It is important to note that this group also had a smaller sample size of 12. Additionally, it is possible that these differences in results are due to the length of hearing aid use, as the prescription hearing aid group had been wearing hearing aids for a longer duration of time (7.89 years vs. 6 months for OTC users), or due to consistency of usage (self-reported hours of hearing aid use: 8.65 h per day for prescription hearing aid users vs. 6.93 h for OTC users at 6 months). While the correlations in unaided hearing loss are reliable, further studies should continue to examine the relationship between DIN and clinical audiology tests in prescription and OTC hearing aid users in larger sample sizes.

### 4.4. Considerations for the Digits in Noise Test

Our results in subjects with untreated hearing loss show that the DIN test is an excellent choice for a remote online test for adults with hearing loss. It shows a significant and meaningful correlation with both puretone audiometry and the QuickSIN clinical test of speech perception in noise, consistent with previous studies [20,21,23,26,27,28,29,30,31,32,33].

In addition to differences in the speech material used (sentences versus digits), the DIN and QuickSIN tests also differ in the background noise utilized. Unlike the QuickSIN test which uses a four-talker babble as the background noise, the DIN test uses a broadband longterm speech-weighted average noise based upon all the included digits. In a study by Wilson and colleagues, multitalker babble and speech-spectrum noise were compared as background noise for the words-in-noise (WIN) test [53]. Their results indicated that both types of background noise were sensitive to distinguishing between those with normal hearing and those with hearing loss. Additionally, those with hearing loss performed similarly in both types of noise, although slightly better in the multi-talker babble. Their study concluded that multi-talker babble may be more appropriate as it may be more representative of everyday listening situations, compared to the speech-weighted noise. In a second study by Wilson and colleagues, sensitivity to four different types of speech perception in noise tests were explored. They found that the QuickSIN and WIN tests, which use multitalker babble, were more sensitive to distinguish between normal hearing and hearing loss participants [54]. However, multitalker babble may introduce more issues regarding test user experience and make independent testing more difficult for individuals [55]. Our results suggest that despite the DIN test using speech-weighted noise, it was able to correctly identify the presence of hearing loss. This suggests that any tradeoffs in sensitivity may not affect the overall validity of the test and may be acceptable to allow for remote testing.

Performing a speech perception test on one’s phone or laptop at home can have many more variables compared to testing in a soundproof booth. Brown and colleagues investigated the performance of the DIN test when performed on a smartphone in the soundfield, comparing the DIN with earphones, smartphone speaker, and loudspeaker in the booth [25]. They found that having the DIN test done on a smartphone through smartphone speakers in a low reverberated room resulted in the same accuracy and reliability as earphone conditions. This is important to keep in mind that highly detailed and accurate instructions should be provided for those planning to test at home, to ensure an appropriate environment for testing in order to get reliable results.

Currently, some versions of the DIN have a cutoff score for what is considered to be normal and could screen with a pass-fail methodology to help determine whether someone should pursue hearing aids. For example, the hearWHO app (offered as a downloadable Android and iOS app), is available for free and utilizes the DIN in this way to allow individuals to self -screen hearing loss and refer on those who do not pass the test [56,57]. Accessibility and ease of availability of this app is an important factor in making an impact on global public health and providing awareness and resources for those with perceived hearing loss.

### 4.5. Study Limitations

There are several limitations to this study. One limitation noted is that the sample sizes of our hearing aid groups were small, and it is possible that this study is underpowered to detect small effects. It is important to note that this data was not gathered for the direct purposes outlined in this study and was a secondary analysis from data gathered from multiple studies. Because of this, there is data that was not collected that could have been useful to analyze for this study. For example, in hearing aid users, we analyzed speech perception in noise using only aided scores rather than benefit scores (i.e., aided vs. unaided), which other studies have done, as we did not have unaided speech-in-noise scores for all participant groups [44]. It is possible that benefit scores may have resulted in differences in correlation strengths and/or significance. Additionally, not all study groups completed the DIN test remotely (i.e., the untreated hearing loss group and prescription hearing aid group), and it would have been useful to compare results across groups as well. Another limitation is that there could be a possible sampling bias, and therefore results may not generalize to a larger population. Because our study required a significant in-person time commitment, this may exclude individuals with full-time work or those unable to take time off or travel to testing sessions.

## 5. Conclusions

In conclusion, our study replicated previous findings that DIN is a useful measure of remote hearing loss assessment and prescription hearing aid validation [20,21,23,26,27,28,29,30,31,32,33]. Our study finds that in untreated hearing loss, the DIN test shows a positive correlation with the QuickSIN test and high frequency puretone averages, indicating its utility as a remote measure of hearing loss that is comparable to current clinical measures. While we did find a correlation between QuickSIN and DIN in our group of prescription hearing aid users, indicating the DIN test’s value for hearing aid patients, this was not significant in our group of OTC hearing aid users. Future studies in OTC hearing aid users are needed to determine whether the DIN test may be a meaningful measure for OTC hearing aid users to validate hearing aid fittings remotely.

## Figures and Tables

**Table 1:** Summary of patient characteristics.

Characteristic	Untreated Hearing Loss (*n* = 46)	Prescription HA (*n* = 15)	OTC HA (*n* = 12)
Age, years, mean ± SD (range)	68 ± 8.4 (46–84)	71 ± 6 (61–79)	70 ± 7.8 (56–81)
Gender, *n* (%)			
Male	19 (41%)	9 (60%)	4 (33%)
Female	27 (59%)	6 (40%)	8 (67%)
Three frequency puretone average (0.5, 1, 2 kHz; PTA-3), dB HL	22.5 ± 8.3 (8.3–44.2)	31.5 ± 12.6 (8.3–52.5)	29.17 ± 7.89 (18.3–44.2)
Four frequency puretone Average (0.5, 1, 2, 4 kHz; PTA-4), dB HL, mean ± SD (range)	28.5 ± 8.4 (11.3–48.8)	38.6 ± 11.4 (16.3–52.5)	35.5 ± 6.3 (26.9–48.8)
High frequency puretone average (3, 4, 6 kHz; PTA-HF), mean ± SD (range)	45.7 ± 13 (20–70)	58.6 ± 12.9 (37.5–87.9)	53.5 ± 6.4 (45.8–61.7)
Duration of hearing aid use in years (prescription HA) or months (OTC HA), mean ± SD (range)	-	7 ± 5.68 (2–23)	5.73 ± 0.47
Daily hearing aid use, hours/day, mean ± SD (range)	-	8.65 ± 5.12 (0.5–16)	6.75 ± 2.71 (0.71–13.55)
Root mean square error (RMSE) in real ear probe measures, mean ± SD (range)	-	5.5 ± 2.5 (2.3–11)	7.5 ± 2.8 (3.9–11.9)

**Table 2:** Summary of speech-in-noise correlations in untreated hearing loss. Correlation results comparing Digits in Noise (DIN) score and QuickSIN score with PTA-3, PTA-4, PTA-HF and WRS (Note: *** = *p* < 0.001; ** = *p* < 0.01; * = *p* < 0.05).

Correlations between Puretone Average and Speech in Noise Testing		
	DIN	QuickSIN
PTA-3	0.33 *	0.35 *
PTA-4	0.49 ***	0.54 ***
PTA-HF	0.65 ***	0.57 ***
WRS	−0.51 ***	−0.47 **

**Table 3:** Speech-in-noise scores over six months of OTC hearing aid use measured in person in the laboratory (*n* = 12).

Measurements in Aided Conditions	QuickSIN Score (dB SNR Loss) (Mean ± SEM)	DIN Score (dB SNR) (Mean ± SEM)
Baseline (i.e., 0 months)	2.54 ± 0.41	1.28 ± 0.41
First follow up (i.e., 1 month)	2.75 ± 0.37	0.93 ± 0.44
Second follow up (i.e., 4 months)	1.86 ± 0.21	1.94 ± 0.47
Third follow up (i.e., 6 months)	2.05 ± 0.31	0.93 ± 0.38

## Data Availability

The data presented in this study are available on request from the corresponding author.
